# Implications of crop model ensemble size and composition for estimates of adaptation effects and agreement of recommendations

**DOI:** 10.1016/j.agrformet.2018.09.018

**Published:** 2019-01-15

**Authors:** A. Rodríguez, M. Ruiz-Ramos, T. Palosuo, T.R. Carter, S. Fronzek, I.J. Lorite, R. Ferrise, N. Pirttioja, M. Bindi, P. Baranowski, S. Buis, D. Cammarano, Y. Chen, B. Dumont, F. Ewert, T. Gaiser, P. Hlavinka, H. Hoffmann, J.G. Höhn, F. Jurecka, K.C. Kersebaum, J. Krzyszczak, M. Lana, A. Mechiche-Alami, J. Minet, M. Montesino, C. Nendel, J.R. Porter, F. Ruget, M.A. Semenov, Z. Steinmetz, P. Stratonovitch, I. Supit, F. Tao, M. Trnka, A. de Wit, R.P. Rötter

**Affiliations:** a CEIGRAM, Universidad Politécnica de Madrid, 28040, Madrid, Spain; b Universidad de Castilla-La Mancha, Department of Economic Analysis and Finances, 45071, Toledo, Spain; c Natural Resources Institute Finland (Luke), 00790, Helsinki, Finland; d Finnish Environment Institute (SYKE), 00251, Helsinki, Finland; e IFAPA Junta de Andalucía, 14004, Córdoba, Spain; f University of Florence, 50144, Florence, Italy; g Institute of Agrophysics, Polish Academy of Sciences, Doświadczalna 4, 20-290 Lublin, Poland; h INRA, UMR 1114 EMMAH, F-84914, Avignon, France; i James Hutton Institute, Invergowrie, Dundee, DD2 5DA, Scotland, UK; j Dpt. AgroBioChem& Terra, Crop Science Unit, ULgGembloux Agro-Bio Tech, 5030, Gembloux, Belgium; k INRES, University of Bonn, 53115, Bonn, Germany; l Institute of Agrosystems and Bioclimatology, Mendel University in Brno, Brno, 613 00, Czech Republic; m Global Change Research Institute of the Czech Academy of Sciences, 603 00, Brno, Czech Republic; n Leibniz Centre for Agricultural Landscape Research (ZALF), 15374, Müncheberg, Germany; o Department of Crop Production Ecology, Swedish University of Agricultural Sciences, Ulls väg 16, 75007, Uppsala, Sweden; p Department of Physical Geography and Ecosystem Science, Lund University, 223 62, Lund, Sweden; q Université de Liège, Arlon Campus Environnement, 6700, Arlon, Belgium; r University of Copenhagen, 2630, Taastrup, Denmark; s Rothamsted Research, Herts, Harpenden, AL5 2JQ, UK; t RIFCON GmbH, 69493, Hirschberg, Germany; u Wageningen University, 6700AA, Wageningen, the Netherlands; v TROPAGS, Department of Crop Sciences, Georg-August-Universität Göttingen, Grisebachstr. 6, 37077, Göttingen, Germany; w Centre for Biodiversity and Land Use (CBL), Georg-August-Universität Göttingen, Büsgenweg 1, 37077, Göttingen, Germany

**Keywords:** Wheat adaptation, Uncertainty, Climate change, Decision support, Response surface, Outcome confidence

## Abstract

Climate change is expected to severely affect cropping systems and food production in many parts of the world unless local adaptation can ameliorate these impacts. Ensembles of crop simulation models can be useful tools for assessing if proposed adaptation options are capable of achieving target yields, whilst also quantifying the share of uncertainty in the simulated crop impact resulting from the crop models themselves. Although some studies have analysed the influence of ensemble size on model outcomes, the effect of ensemble composition has not yet been properly appraised. Moreover, results and derived recommendations typically rely on averaged ensemble simulation results without accounting sufficiently for the spread of model outcomes. Therefore, we developed an Ensemble Outcome Agreement (EOA) index, which analyses the effect of changes in composition and size of a multi-model ensemble (MME) to evaluate the level of agreement between MME outcomes with respect to a given hypothesis (e.g. that adaptation measures result in positive crop responses). We analysed the recommendations of a previous study performed with an ensemble of 17 crop models and testing 54 adaptation options for rainfed winter wheat (*Triticum aestivum* L.) at Lleida (NE Spain) under perturbed conditions of temperature, precipitation and atmospheric CO_2_ concentration. Our results confirmed that most adaptations recommended in the previous study have a positive effect. However, we also showed that some options did not remain recommendable in specific conditions if different ensembles were considered. Using EOA, we were able to identify the adaptation options for which there is high confidence in their effectiveness at enhancing yields, even under severe climate perturbations. These include substituting spring wheat for winter wheat combined with earlier sowing dates and standard or longer duration cultivars, or introducing supplementary irrigation, the latter increasing EOA values in all cases. There is low confidence in recovering yields to baseline levels, although this target could be attained for some adaptation options under moderate climate perturbations. Recommendations derived from such robust results may provide crucial information for stakeholders seeking to implement adaptation measures.

## Highlights

• Crop model ensemble size and composition affect the ensemble outputs.
• Recommendations on adaptation are sensitive to model ensemble composition and size.
• The new EOA index effectively measures the confidence level of recommendations.
• Effective adaptation of wheat in the Mediterranean is feasible with high confidence.
• The EOA index can be applied to assess confidence in many other contexts.

## 1 Introduction

In the absence of effective mitigation and adaptation, future climate change is expected to have adverse effects on crop production in many regions of the world, with the risk of severe impacts increasing with continued warming after 2050 (Asseng et al., 2015; IPCC, 2014). For cereals, some studies report future yield decreases in southern Europe (Kovats et al., 2014). Adaptation will be crucial to reduce such negative impacts, maintain or even enhance levels of crop production (Challinor et al., 2014; Lobell, 2014). Crop models, by representing some of the key interactions between crops, their environment and their management (Rötter et al., 2011) can be useful tools for evaluating some of the available options for field-level adaptation of crop production (Rötter et al., 2015; Wallach et al., 2014).

The use of several different models in a multi-model ensemble (MME) to quantify aspects of uncertainty in model simulations has been a practice employed in climate modelling over several decades (see, e.g. Knutti, 2010), but has only recently been adopted in agricultural assessment (Asseng et al., 2015; Bassu et al., 2014; Li et al., 2015; Palosuo et al., 2011; Wallach et al., 2016; Yin et al., 2017). Results from MMEs have been reported as offering more robust information than from any individual model member (Asseng et al., 2015; Iocola et al., 2017; Martre et al., 2015; Palosuo et al., 2011; Rötter et al., 2012; Yin et al., 2017).

MMEs have also been used to construct impact response surfaces (IRSs) plotted from the result of sensitivity analyses (Fronzek et al., 2018; Pirttioja et al., 2015). IRSs assess the response of an impact variable to systematic perturbations of two explanatory variables (typically precipitation, P, and temperature, T). A further step has been to plot the difference between impacts simulated with and without adaptation in response to joined P and T changes as adaptation response surfaces (ARSs; Ruiz-Ramos et al., 2018). These have been presented as ensemble-averaged results, providing a measure of the potential effectiveness of the adaptation. However, adaptation effectiveness can vary considerably among models, so the average offers little information about the confidence that might be attached to that result for making a recommendation. Such a measure could be quite useful for guiding an adaptation decision.

Let us consider the “outcome agreement” as a potential indicator for characterizing this confidence (i.e. the level of consistency and agreement between model outcomes; Stainforth et al., 2007). It can be defined as a combination of two components: first, the degree of ensemble consensus on a given hypothesis to be tested, and second, a measurement of disagreement between ensemble members relative to the hypothesis. In this context, by linking a recommendation to hypothesis compliance, the measure of outcome agreement becomes an indicator of confidence based on the available information. This is distinct from uncertainty, which is often related to an estimate of the spread of that information. However, uncertainty is still present and affects the levels of outcome agreement.

In previous MME analyses, little attention has been paid to how an ensemble is built (exceptions include Fronzek et al., 2018; Li et al., 2015; Yin et al., 2017) and how this may have affected the ensemble results. Previous studies have mostly analysed ensemble size to examine convergence of outcomes and how this may have affected the results from ensemble modelling. For instance, Martre et al. (2015) used a 27-member wheat MME and tested the accuracy of results against observations for sub-ensembles of varying sizes, focusing on defining a minimum ensemble size. They found that adding more than eight members to a MME did not significantly improve accuracy of results. Li et al. (2015) and Yin et al. (2017) used a MME to measure the uncertainty of predicted current yield by comparing model means for ensembles of all possible sizes and composition with observations. Obviously, this approach cannot be used under future scenarios for which there are no observations.

Even in the absence of observations with which to compare, some estimates of future outcomes may also be evaluated qualitatively. For instance, some outcomes may be excluded from further consideration because they are judged implausible, based on the knowledge and experience of experts familiar with crop responses in different environments. Hence, by devising a set of plausibility criteria, it may be possible to define minimum requirements to be met by the members of a MME. One such example is the AOCK (“according to our current knowledge”) concept, which was introduced by Ruiz-Ramos et al. (2018) in a study using simulation outputs from a 17-member wheat MME to evaluate different adaptation options in Lleida (north-eastern Spain). They defined a list of criteria for excluding members with results that appeared implausible based on our current knowledge. This resulted in ensembles of different size and composition (depending on which models were excluded) for each adaptation option considered.

Few previous crop modelling studies have analysed in detail how the recommendations derived from MME simulations could be affected by the ensemble composition and size (e.g. some aspects are analysed in Rosenzweig et al., 2014). This is a major issue that limits the applicability of results, especially when no observations are available to evaluate the predictive skill of the MME-based results, as is the case for yields under a perturbed climate. Furthermore, crop MMEs used so far are largely “ensembles of opportunity” (Tebaldi and Knutti, 2007; Wallach et al., 2016) – participating model runs are often determined by volunteer contributions from crop modelling groups.

To address this, the objective of this paper is to assess the effect of ensemble size and composition on the ensemble outcome agreement, and therefore on the confidence of the derived recommendations from MME results. By supplementing a recommendation for an adaptation option with an estimate of its confidence, the usefulness of the information for stakeholders may be much enhanced. Therefore, the specific objectives of this study are: 1) to develop an index for assessing outcome agreement of MME results, and 2) to illustrate an application of the index by analysing the confidence of MME-derived adaptation recommendations reported by Ruiz-Ramos et al. (2018).

## 2 Material and methods

### 2.1 Study case

The adaptation study performed by Ruiz-Ramos et al. (2018) was used as a study case to illustrate the method of development and application of the proposed index. Details about the study site, experimental and climate data, calibration process, sensitivity analysis, simulated adaptation options and ensemble building can be found there; only a general overview is provided in this section.

The study site was Lleida, located in the northeast of Spain, within the “Mediterranean South” environmental zone depicted in Metzger et al. (2005). A 17-member crop model ensemble was used in this study, comprising 14 crop models and 17 independent simulation sets (see Table S1, supplementary material). Two modeller groups used CERES-wheat and three of them used WOFOST, but given the different methods used to calibrate and set up each model, they were considered as independent ensemble members, following Pirttioja et al. (2015), as the differences between differently calibrated CERES/WOFOST members were similar to those between other crop models.

Models were calibrated for winter wheat (*Triticum aestivum* L. cv. Soissons) using observed phenological (flowering and maturity) dates and biomass and yield data from field experiments conducted in the study area during the 2003–2004 and 2005–2006 growing seasons (Abeledo et al., 2008; Cartelle et al., 2006). An atmospheric carbon dioxide concentration of 360 ppm was assumed. The calibration performance of all models was categorised as “good” following evaluation criteria reported by Jamieson et al. (1991). Two soil profiles – shallow and deep – representing actual conditions were selected (Fig. 1b in Ruiz-Ramos et al., 2018), with different depth, texture and water holding capacity (126 mm *vs*. 290 mm in the shallow and deep soils, respectively).

Daily maximum and minimum air temperature, precipitation, solar radiation, humidity and wind speed observations for the period 1981–2010 from the AEMET station at Lleida were selected as baseline input data for the model simulations. Daily T and P data were then perturbed to perform a sensitivity analysis, using a “change factor” approach with a seasonal weighting based on the ensemble mean pattern of projected seasonal change from Harris et al. (2010), whilst preserving annual mean change intervals (Fronzek et al., 2010). T was modified between −1 °C and +7 °C at 1 °C intervals, and P from -40% to +30% at 10% intervals, resulting in 72 perturbation combinations that covered the spread of projections for Spain by mid-century for the SRES A1B emission scenario (Harris et al., 2010). Relative humidity was assumed to remain unchanged from the baseline, requiring adjustments to vapour pressure and dew point for those models using these as inputs. Two levels of [CO_2_] representing two 20-year time slices for periods centred on 2030 and 2050 according to SRES A1B projections (IPCC, 2000) were considered (447 ppm and 522 ppm, respectively). Other variables were kept at baseline levels.

The study adopted a total of 23 adaptation options (each comprising one or more simulated action) that were found to have a positive response (i.e. yield increases when adaptation is simulated relative to an unadapted simulation) out of the 54 options tested. Selected adaptation options comprised changes in vernalisation requirements, adopting cultivars with shorter and longer phenological phases, advancing the sowing date by 15 days, and applying supplementary irrigation (40 mm during flowering). Full irrigation was also included to provide a reference for the optimal productive potential. MME outcomes from all possible P and T perturbation combinations for both 447 ppm and 522 ppm [CO_2_] and for both shallow and deep soils were adopted. See Ruiz-Ramos et al. (2018) for a full description of the adaptation options and how these were simulated.

### 2.2 Response surface analysis

An impact response surface (IRS) consists of a plotted surface that depicts the response of a studied variable (e.g. crop yield) to changes in two explanatory variables (e.g. P and T). An adaptation response surface (ARS) plots the difference between yield responses with and without adaptation being considered, usually as a percentage change. This metric is defined as the “adaptation value”. It measures the effect of adaptation under a given combination of perturbation of T, P and [CO_2_] compared to no adaptation under the same perturbations. A second metric, labelled “recovery value”, is the relative difference between the yield response including an adaptation option and the baseline yield response (i.e. for an unperturbed simulation, 360 ppm of [CO_2_] and unadapted management). The “recovery value” measures the ability of an adaptation option to maintain the yields of the baseline simulation under unperturbed conditions.

In Ruiz-Ramos et al. (2018), the ensemble median of every perturbation combination was used to construct IRS and ARS surfaces, which were subsequently analysed to compare adaptation performance and make recommendations. This study analyses the underlying data that were used to construct the surfaces to provide additional information regarding the confidence of the recommendations.

### 2.3 Exploring possible ensembles

The method presented here involves testing the hypothesis that an adaptation option is effective (adaptation value higher than an user-defined threshold), though it could be generalised to test other hypotheses. It comprises several steps: 1) computing the ensemble median of adaptation and recovery values for all P and T perturbations and combinations of ensemble members for all ensemble sizes and compositions, 2) calculating an index to measure the agreement between the ensemble outcomes for each individual adaptation option and for every P and T perturbation, and 3) interpreting the index to assess the confidence of recommendations and derive conclusions. One feature sought for the index was to make use of two metrics that are conventionally used to report ensemble results: the average or aggregated response (e.g. mean or median) and the minimum ensemble size required to produce a given outcome. In this way, the index might conceivably be compared to earlier studies that use MME outputs.

#### 2.3.1 Processing of ensemble combinations

**Fig. 1:**
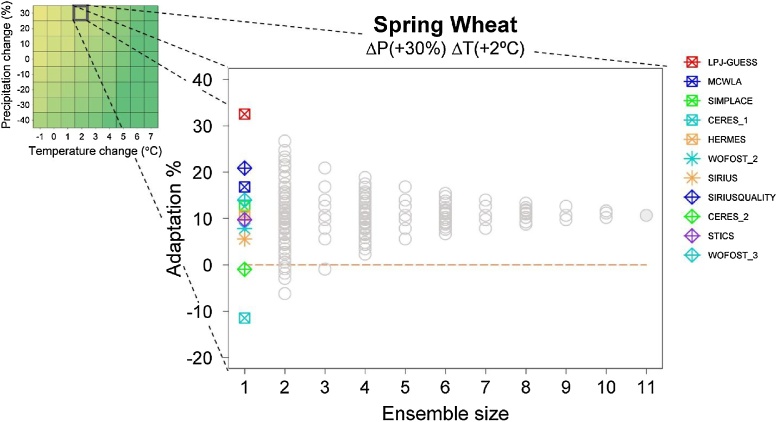
Adaptation responses (% change in yield) following a switch from a winter to a spring cultivar for a specific perturbation within the adaptation response surface (T change of +2 °C and a P change of +30%). Adaptation values are shown for different ensemble sizes up to the maximum number of available model outputs for this adaptation option (11). Ensemble size of 1 shows results from individual ensemble members indicated by different symbols and colours. Each grey circle represents the median of the 30-year averaged results for all combinations of ensemble composition and size. Ensemble size 11 shows the median of the 30-year period (1980–2010) averaged results used in Ruiz-Ramos et al. (2018).

Ensemble composition is defined here as the specific combination of models contained in the ensemble, while ensemble size is the number of models comprising a specific combination. The aggregation method for the MME outputs consists of an algorithm used to obtain a single value that will represent the whole MME. Regardless of whether the aggregation method is a simple one (e.g. using mean or median) or a complex one (e.g. using weights), the index proposed here can be calculated in the same way. In this study, the chosen aggregation method was the median, so ensemble medians of adaptation and recovery values for all T and P perturbation combinations were computed for every possible ensemble composition and size. An example for one P and T perturbation and an adaptation option that involves switching from a winter to spring wheat cultivar (an adaptation for which 11 model outputs were available) is depicted in Fig. 1.

The maximum ensemble size in the study case is 17, which is the total number of models in the ensemble. So, for all 17 models, the maximum number of different combinations of ensemble composition and size (i.e. subsets of the full ensemble) is expressed by:

$$\sum_{i=1}^{17}C_{17,i}=131071$$

where $C_{\text{17,}i}$ represents the binomial coefficient indicating the number of combinations of 17 elements taken from *i* in *i*.

#### 2.3.2 Index of ensemble outcome agreement (EOA)

Let us define a hypothesis (H) that the value to be tested (e.g. adaptation or recovery value) should be greater than a given threshold (e.g. > 0% for the simplest case implying a yield increase relative to the yield of the unadapted or baseline simulation). The index of Ensemble Outcome Agreement (EOA) for a given perturbation combination of T and P, using a specific aggregation method (e.g. median or mean), is a measure of confidence that the ensemble outcome fulfils the hypothesis (i.e. that H is true) according to the available information (in this case, the available crop simulations for a particular adaptation option).

Once the hypothesis is established and the aggregation method chosen, the EOA is calculated using Eq. 1:

(1) $$EOA=\frac{1-\frac{ES-AF}{N+1}}{1-\frac{1}{N+1}}$$

where *N* is the maximum ensemble size, dependent on the available models for the selected adaptation option to be tested (e.g. *N* = 11 in Fig. 1); *ES* is the minimum ensemble size for which all ensemble combinations fulfil H (e.g. *ES* = 4 in Fig. 1). If no sub-ensemble fulfils the condition, then EOA = 0. *AF* is an adjustment factor within the interval [0,1] (see below, Eq. 2). Note that *N* is not always the total number of models of the ensemble (17 in this study) because not all crop models simulated all adaptations options.

The EOA has a value within the interval [0,1] and it deviates from zero only when the available information indicates that it is more likely than not that H is fulfilled. So, if the selected aggregated metric does not fulfil H, then the EOA will be 0, regardless of whether individual members show a positive response. For instance, suppose there are four values, two of them fulfilling H and the other two not, with both pairs of values at identical distances either side of the threshold value. In that case, based on the available information, we cannot tell if it is more likely that H is fulfilled or not, and the assigned EOA value will be 0, even though two values fulfilled H.

**Fig. 2:**
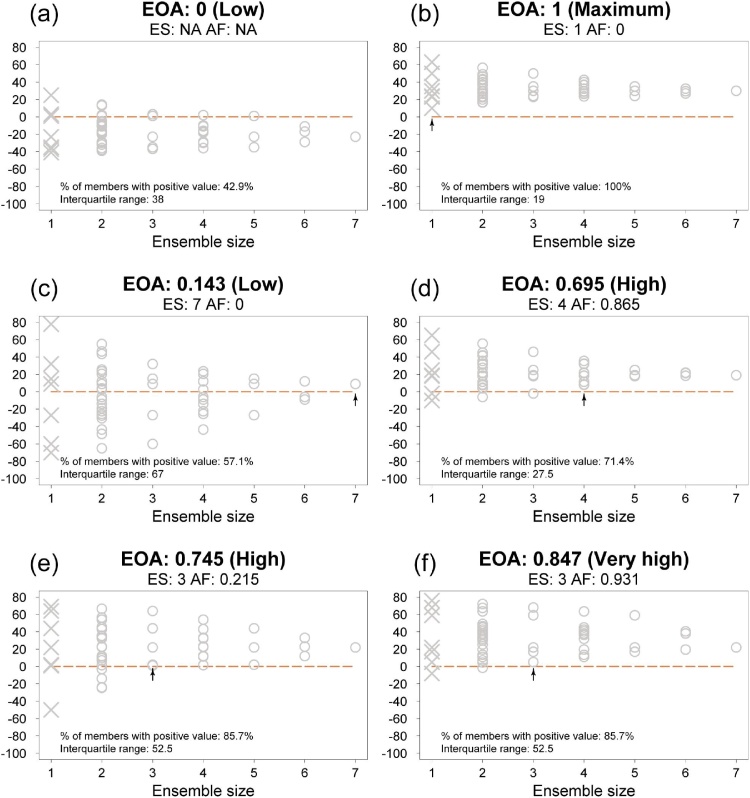
Examples of hypothetical multi-model ensemble (MME) outcomes (vertical axis) for different ensemble sizes (horizontal axis). Size 1 indicates outcome from individual members (crosses). Medians of all combinations of MME for each size are shown as open circles. Panels show: at top, values and class of Ensemble Outcome Agreement (EOA), minimum ensemble size for which all permutations fulfil the hypothesis (*ES*) and adjustment parameter (*AF*), and at bottom-left, the proportion of members giving positive values and the interquartile range (IQR) of the full MME. The hypothesis tested was that the MME median is greater than 0. See Table 1 for interpreting EOA classes. The arrow depicts the minimum ensemble size (*ES*) for which every possible ensemble composition result is larger than 0.

Fig. 2 illustrates some hypothetical MME outcomes and their associated EOA metrics. EOA is 0 when there is no ensemble size for which H is fulfilled by all combinations of ensemble members; hence *ES* is undefined (e.g. Fig. 2a). EOA is 1 if every possible ensemble fulfils H (e.g. Fig. 2b). EOA is greater than 0 when H is fulfilled for at least the largest ensemble size (e.g. Fig. 2c). The closer EOA is to 1, the greater the outcome agreement and the confidence about H being fulfilled.

There can be large differences in the EOA depending on the value of *ES* (e.g. compare Fig. 2c and d). Even if around half of the possible sub-ensemble medians are above the threshold used to define H, as in Fig. 2c, the index is close to 0 because the other half are below the threshold. For the same *ES*, the index can be different depending on the spread among the ensembles for smaller sizes up to *ES*-1 (e.g. compare Fig. 2e and f). For that reason, we have introduced *AF* (Eq. 2), which is an adjustment factor for distinguishing situations with the same *ES* but with different outcome agreement due to different ensemble spread regarding the threshold. *AF* is estimated by calculating the agreement-disagreement ratio of combinations of ensemble members, for ensemble sizes lower than *ES*, regarding H (defined by the threshold and the result of member combinations), and it is calculated as follows:

(2) $$AF=\max\left(1-\left(\frac{ES-1}{\sum_{i=1}^{ES-1}\frac{my_{i}}{mn_{i}}}\right),0\right)$$

where $my_{i}$ is the mean absolute distance to the threshold of every value fulfilling the hypothesis for ensemble size i (if no value is found then $my_{i}$ is 0); $mn_{i}$ is the mean absolute distance to the threshold of every value not fulfilling the hypothesis for ensemble size i (if no value for $mn_{i}$ is found then the $my_{i}/mn_{i}$ ratio will be the maximum one found for other ensemble sizes).

The more (less) demanding the threshold, the lower (higher) will be the expected number of ensemble subsets fulfilling H (ensemble medians and individual members shown in Fig. 1, Fig. 2), the closer to 0 (1) will be AF, and the lower (higher) the resulting value of EOA.

It is important to note that the EOA is not the probability of H being true; rather it assigns values close to 0 to situations with a very low level of outcome agreement, and values close to 1 to situations with high outcome agreement according to the available ensemble members. A low EOA value indicates a lack of reasonable agreement of the models regarding the fulfilment of H, but it does not necessary imply a greater spread among models. For instance, the ensemble members may present values that are clustered around the threshold (small spread) but with some members above and others below (low agreement). Furthermore, the low EOA value itself cannot be used for explaining the reasons for not fulfilling H; rather it may hint at outcomes that may be candidates for a more in-depth analysis to try to understand if the low values are due to the spread of results, to the selected threshold, or both.

EOA values are classified according to an intuitive interpretation system in Table 1 (fixing the adjustment factor, *AF*, at 0 to facilitate understanding). EOA intervals were defined seeking simple relationships between values of *ES* and *N*. EOA classes were chosen based in part on language used for confidence characterisation in the IPCC uncertainty guidance document (Mastrandrea et al., 2010).

**Table 1:** EOA classes and ranges of values with interpretations of some EOA values (for *AF* = 0).

EOA range	EOA class	EOA value (with *AF* = 0)	Interpretation	*ES*
[0, 0.25)	Low	0	The aggregated ensemble value does not fulfil the hypothesis. No ES is found.	n/a
[0.25, 0.5)	Medium	0.25	The minimum size for which all combinations fulfil H is three-quarters of the available members plus one	(3/4)*N*+1
[0.5, 0.75)	High	0.5	The minimum size for which all combinations fulfil H is half of the available members plus one	(*N*/2)+1
[0.75, 1)	Very high	0.75	The minimum size for which all combinations fulfil H is a quarter of the available members plus one	(*N*/4)+1
1	Maximum	1	All available members fulfil the hypothesis	1

EOA: ensemble outcome agreement; *AF*: adjustment factor; *ES*: minimum ensemble size for which all permutations fulfil the hypothesis H; *N*: ensemble size.

Other simpler indices, such as the proportion of all members fulfilling the hypothesis, may lead to an overestimation of the outcome agreement value (see “% of members with positive value” in Fig. 2c). Measures of spread, such as the interquartile range, together with the proportion of members fulfilling the hypothesis and with the final averaged values are not able to discriminate situations with a different EOA class (Figs. 2e *vs.* f).

The EOA was computed for every possible perturbation of T and P considered in Ruiz-Ramos et al. (2018). EOA results were examined either by analysing many adaptation options at once, to identify adaptation options with the highest outcome agreement, and by focusing on one particular adaptation option using response surfaces. The latter facilitate identification of those T and P perturbations (i.e. the regions of the response surface) for which the ensemble members agree more on their response to a given adaptation. In addition, for illustrative purposes, an individual perturbation combination (i.e. “grid box” on the plot) was selected to demonstrate the analysis of underlying data that was carried out across all perturbation combinations. R code for EOA computation is available as supplementary material.

## 3 Results

In this section, adaptation, recovery and their respective EOA values are analysed in depth for the most unfavourable conditions of the simulated data set (i.e. 447 ppm of atmospheric [CO_2_] and shallow soil). Results for contrasting conditions (552 ppm of atmospheric [CO_2_] and deep soil) can be found in supplementary material (Figs. S1 to S3).

**Fig. 3:**
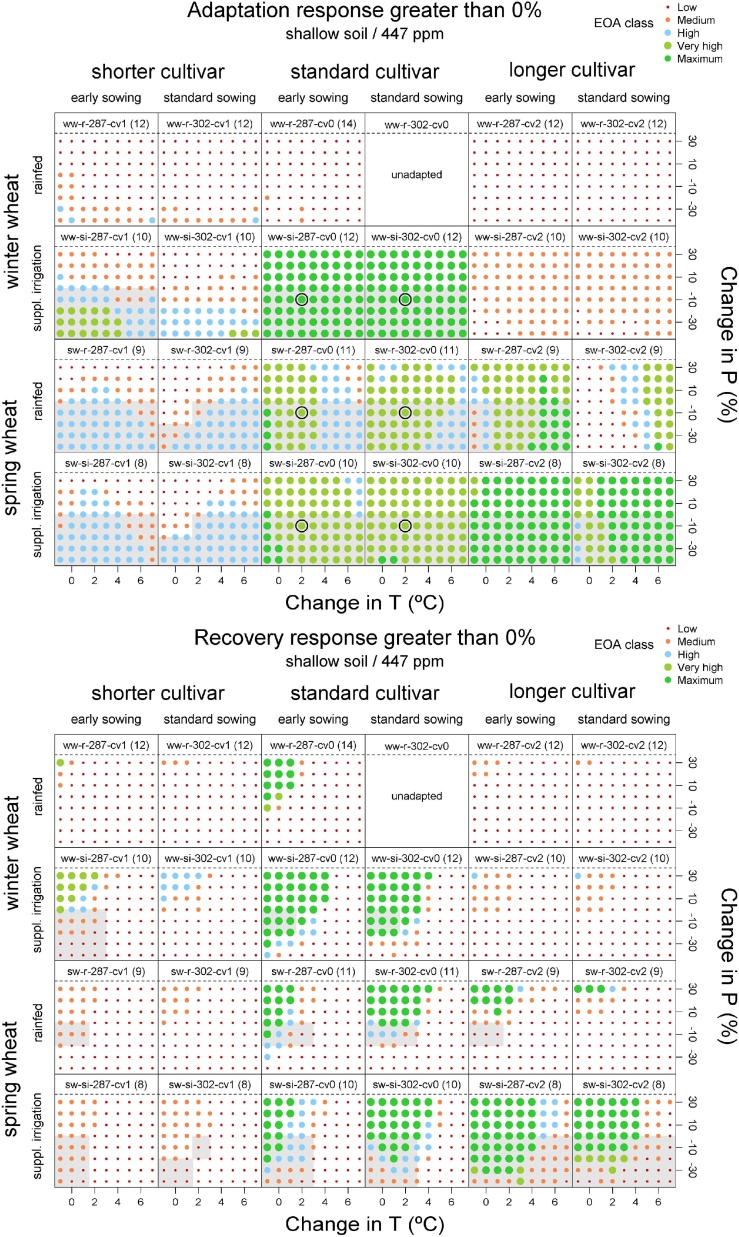
Ensemble outcome agreement (EOA) concerning a positive adaptation response (upper panels) and a positive recovery response (lower panels) for the most promising adaptation options from Ruiz-Ramos et al. (2018), assuming shallow soil and [CO_2_] of 447 ppm for different temperature (T, ºC) and precipitation (P, %) perturbations. Rows of panels: winter wheat (top two) and spring wheat (bottom two), each for rainfed (upper) and 40 mm of supplementary irrigation applied at anthesis (lower). Columns of panels from left to right are paired by growing duration (10% shorter, standard and 10% longer), each pair alternating between early (DOY, 287) and standard sowing dates (DOY, 302). Grey-shaded areas of each subplot indicate the T and P perturbations for which the adaptation option was recommended in Ruiz-Ramos et al. (2018) (P increases not considered). Codes for 23 adaptation options and the unadapted option are described in Table S2 and shown in panel headers with the number of ensemble members used in parentheses. EOA classes are described in Table 1. Black open circles highlight the cases analysed in Fig. 4.

EOA values for those adaptation options simulated in Ruiz-Ramos et al. (2018) that showed some adaptation and/or recovery potential (23 out of 54 options) are shown in Fig. 3. Both single and combined adaptation options were considered.

### 3.1 Adaptation response

The highest values of EOA across the perturbation ranges were found for winter wheat applying supplementary irrigation (combined adaptation ww-si). The classification into different EOA classes for ww-si was found to depend on the duration of the growing cycle; longer duration cultivars (cv2) showed low and medium EOA, while values of EOA for the shorter duration cultivars (cv1) ranged from low to very high. Standard duration cultivars (cv0) were the best choice to obtain the maximum EOA value (1) for every perturbation (Fig. 3).

Adaptation options using spring wheat (sw) showed very high EOA values for standard duration cultivars (cv0) and standard or early sowings for the majority of the analysed perturbations (medium-high EOA level for severe warming). If supplementary irrigation is applied (sw-si) confidence in the results using EOA is very high or greater for almost every perturbation when using standard and long duration cultivars, and standard and earlier sowing dates (Fig. 3).

Values of EOA that were very high (close to 1) or maximum (EOA = 1) were found in every tested perturbation for the following four combinations of adaptation options (when not indicated, cultivar type, cycle length and sowing are standard options): supplementary irrigation (ww-si-302-cv0), supplementary irrigation and early sowing (ww-si-287-cv0), spring wheat with supplementary irrigation (sw-si-302-cv0) and spring wheat with supplementary irrigation, longer cultivar and early sowing (sw-si-287-cv2).

Adaptation EOA values were similar for the two analysed [CO_2_] levels. In general, the adaptation EOA value was greater for shallow soil than for deep soil (see Figs. S1 to S3 in supplementary material) as more models agreed in detecting more intense water stress in the shallow soil than in the deep one.

### 3.2 Recovery response

For adaptation options without irrigation and P decreases, high EOA values were found for spring wheat, mostly combined with early sowing and standard cultivar (sw-r-287-cv0), with some examples for winter wheat with early sowing. When P decreases are combined with T increases of 2 °C or greater, no adaptation option showed high EOA levels when irrigation is not available. Most EOA values classified as at least high were found for P increases. As expected, EOA values for the same adaptation option were enhanced as P increased.

For adaptation options with supplementary irrigation, standard (si-302) or early sowings (si-287) showed high EOA levels for P decreases and up to 3 °C of warming. Highest EOA values were found for the standard and longer cultivars mostly up to T increases of 4 °C. The combination of spring wheat, longer cultivar and early sowing (sw-si-287-cv2) offered the highest values across the range of T changes out of all options, with high EOA values even for a T increase of 6 °C and severe P decreases (Fig. 3).

EOA values for recovery response showed some increase with higher [CO_2_], especially with decreases in precipitation. The EOA values for recovery response were more sensitive to changes in CO_2_ levels and soil type than EOA for adaptation response (see Figs. S1 to S3 in supplementary material).

### 3.3 EOA analysis example for a grid box

**Fig. 4:**
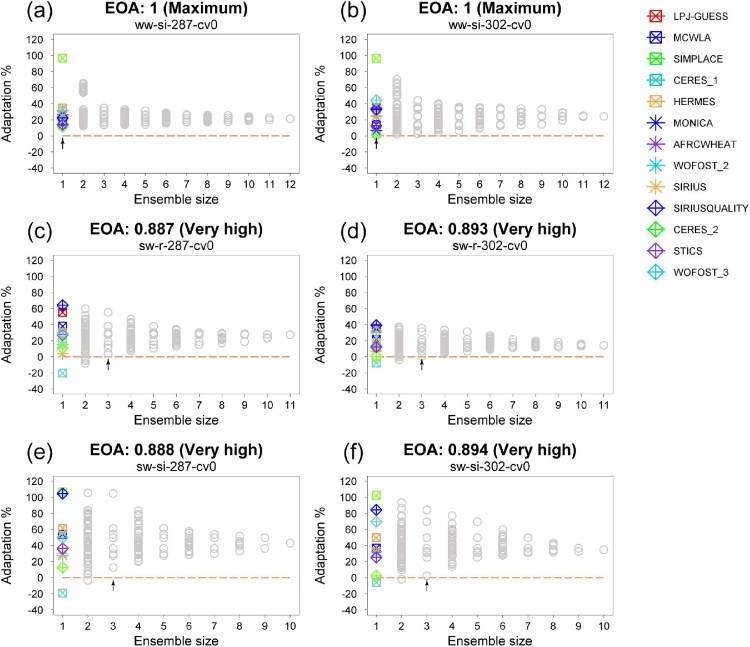
Multi-model ensemble responses and resultant values of ensemble outcome agreement (EOA) for a single (illustrative) perturbation combination, T+2 °C/P-10% for a shallow soil and [CO_2_] of 447 ppm. Options: a) and b) supplementary irrigation (si), c) and d) rainfed spring wheat (sw), e) and f) spring wheat with supplementary irrigation (sw-si), a), c) and e) for early (day-of-the-year 287) and b), d) and f) standard (302) sowing dates. For all cases, a standard cultivar (cv0) was simulated. Ensemble size of 1 shows 30-year averaged results from individual ensemble members indicated by different symbols and colours. Grey circles represent the medians of different sub-ensembles. The hypothesis tested was that the adaptation response is greater than 0%. See Table 1 for interpreting EOA classes. An arrow shows the minimum ensemble size for which every possible ensemble composition result is larger than the adaptation threshold (0% in this example). Codes for adaptation options are described in Table S2. The adaptation options for the considered perturbation analysed here are highlighted in Fig. 3 by black open circles.

An example cell (T+2 °C/P-10%) was selected to illustrate the analysis made for every perturbation. EOA values are broken down into their constituent elements in Fig. 4 (cases analysed in Fig.4 are highlighted in Fig. 3 with a black open circles). Every member of the ensemble (of 12) showed a positive adaptation response to supplementary irrigation (si) for earlier and standard sowing dates (Fig. 4a and b, respectively). A switch to spring wheat (sw) produced a negative adaptation response in only one ensemble member under rainfed conditions, with very high EOA values for early (Fig. 4c) and standard (Fig.4d) sowing dates, the latter showing adaptation values with a fairly low spread, converging on +15% as the MME size increased. Adding supplementary irrigation (sw-si) widened the spread towards higher positive adaptation responses but did not much affect the lower end, with still one ensemble member producing a negative response. EOA values remained at a very high level (Fig. 4e and f).

**Fig. 5:**
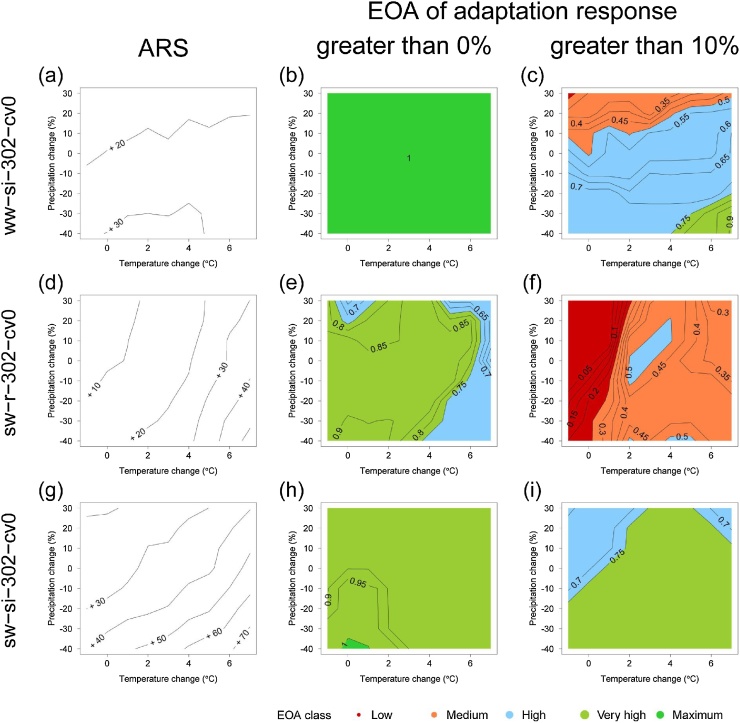
Response surfaces with respect to changes in annual mean temperature and precipitation for the ensemble median adaptation response (left column) and ensemble outcome agreement (EOA) for adaptation responses greater than 0% (centre column) and greater than 10% (right column). Adaptation options shown are a)-c) supplementary irrigation (si); d)-f) substitution from winter to spring wheat (sw) and g)-i) spring wheat with supplementary irrigation (sw-si). Simulations were for shallow soil and [CO_2_] of 447 ppm. See Table 1 for interpreting EOA classes. Codes for adaptation options are described in Table S2.

The variation of ensemble average adaptation responses and their respective EOA values across different climates can be portrayed using response surfaces. Median adaptation responses are shown for three adaptation options as adaptation response surfaces (ARS) in Fig. 5 (left hand plots), including one rainfed option. The confidence in these changes is represented in Fig. 5 using EOA surfaces for two hypotheses of adaptation responses: exceeding thresholds of 0% (i.e. increased yields compared to the unadapted simulation) and 10% (i.e. > 10% yield increase). Plots for the supplementary irrigation (ww-si) adaptation option show positive adaptation response (Fig. 5a) with maximum EOA for the 0% threshold and for every perturbation combination (Fig. 5b). Isolines are predominantly horizontal for the 10% threshold (Fig. 5c) indicating that the outcome agreement for that threshold was mainly linked to precipitation change: the greater the decline in P, the higher the EOA value. As would be expected, EOA values decrease as the adaptation response threshold increases – in other words, confidence in fulfilling the hypothesis H declines as H becomes more demanding to achieve (e.g. compare Fig. 5e with Fig. 5f). EOA values were high or very high in almost every perturbation combination when using the spring wheat (sw) adaptation option with threshold 0% even under rainfed conditions (Fig. 5e), and at least medium for the 10% threshold for severe drying and warming (Fig. 5f). If supplementary irrigation is used with spring wheat (sw-si), EOA was very high for both thresholds (Figs. 5h and i). The patterns of variation in EOA values differed from those of the ARS. For instance, for large increases in P, median responses to the spring wheat (sw) adaptation option showed increases from 10% to 30% with higher T (Fig. 5d), but EOA values show a weak increase and then a stronger decline across the same climate perturbations for the 0% threshold (Fig. 5e).

## 4 Discussion

### 4.1 The need for alternative metrics describing multi-model ensemble outcomes

In recent years, the crop model ensemble approach has been used in many studies, in general obtaining the MME outcome by averaging the individual member results using the mean (e.g. Iocola et al., 2017; Palosuo et al., 2011; Rötter et al., 2012; Yin et al., 2017) or the median (e.g. Asseng et al., 2015; Bassu et al., 2014; Martre et al., 2015; Pirttioja et al., 2015). In our study the median was selected as it present advantages for small ensemble sizes or for ensembles where a single model member that is clearly biased has a disproportionate influence on the mean (e.g. as shown by Rötter et al., 2012 and Wallach et al., 2018). However, the influence of switching from median to mean in our results was also analysed, resulting in a low impact on the EOA values (an example is given in supplementary material Fig. S4), in agreement with Martre et al. (2015) where mean and median displayed a similar behaviour.

In terms of size, some MME studies have attempted to analyse the minimum number of models required to obtain reliable results (Bassu et al., 2014; Maiorano et al., 2017; Martre et al., 2015). Maiorano et al. (2017) demonstrated that the suggested minimum ensemble size (*ca.* 10 models) proposed in Martre et al. (2015) could be reduced if the quality of crop models could be improved, suggesting that a given ensemble size might not guarantee high quality in the results. A logical consequence would be that if a new ensemble of models not previously tested were to be constructed, reliance on the criterion of minimum ensemble size would seem to be at best uncertain and possibly misleading, implying that further analysis of the MME composition would be required (see, e.g. Knutti, 2010).

This study demonstrates that the final averaged result can vary widely depending on the composition and size of the MME and we would argue that using an aggregate summary value representing the full ensemble is not sufficient for representing the diversity of information offered by MME results. Alternative metrics like the EOA index proposed here are required to complement the existing MME averages.

The EOA index provides insight beyond the expected averaged yield response, judging the level of agreement of MME results in relation to a hypothesis concerning the yield response of interest. Among the parameters of the EOA index, *ES* is the one that most affects its value. Nevertheless, an adjustment factor *AF* is also required to distinguish situations with the same ES value but where different EOA values should be assigned due to internal differences in ensemble behaviour. The adjustment factor *AF* also produces an EOA index that is continuous, whereas the use of the *ES* parameter alone would have resulted in a discrete index with the number of possible values depending on the number of ensemble members.

A high EOA value occurs in situations where most members fulfil the hypothesis. Importantly, a large averaged adaptation response does not necessarily imply a large index value. In this sense, when seeking a more robust outcome it would be preferable to obtain a low adaptation response with high EOA rather than a high adaptation response with low EOA. The latter can occur when the averaged response is positive and large, yet not all ensemble members suggest that the hypothesis is fulfilled. This situation can appear when the ensemble spread is large. Moreover, for a given positive adaptation response, higher EOA values were found for a threshold of 0% than for 10% (and results not shown for a threshold of 20% confirm this). This implies that models agree more in adaptation sign (positive or negative) than in adaptation value (specific % of change when the adaptation is applied) as was previously suggested by Ruiz-Ramos and Minguez (2010).

The EOA index provides information that is potentially useful for decision making, as it relates to a hypothesis that could be defined by stakeholders. Other simpler indices, such as the ratio of ensemble members fulfilling the hypothesis, or measures of spread such as the interquartile range (IQR) or coefficient of variation (e.g. Pirttioja et al., 2015) do not effectively provide information on confidence of the hypothesis made based on the ensemble. Those indices can be challenging to interpret for stakeholders, because 1) IQR does not relate to a critical threshold and 2) they still need to be interpreted together with the ensemble average. EOA has the advantage that it combines information of the ensemble average and ensemble spread in relation to a critical threshold into a single index. Use of the EOA index (rather than just the final averaged ensemble result based on means or medians) could be of great relevance for informing adaptation assessment because the interpretation of results and derived recommendations can be affected dramatically.

### 4.2 Evaluating the EOA index in appraising adaptation options for wheat in north-east Spain

The utility of the EOA index has been tested here using MME adaptation responses reported for wheat yields in north-east Spain by Ruiz-Ramos et al. (2018). Many more adaptation options were found to produce positive responses (enhanced yields) with high EOA values across a range of climates than were able to return yields to their baseline levels (recovery response), for which only a few adaptation options under a limited number of perturbations showed a high EOA level. Thus, our study indicates that the feasibility of obtaining a positive yield response does not exclusively rely on water availability for supplementary irrigation, in contrast to yield recovery, for which there are high EOA values only for options including supplementary irrigation.

More specifically, rainfed wheat based adaptation options could be problematic because the EOA value for a positive adaptation response to winter cultivars was zero for almost every climate perturbation except moderate wetting. The bright side is that for every perturbation it was possible to find at least one option with positive adaptation response and a high EOA value when switching to spring wheat (even without supplementary irrigation). These results and their index values are consistent with the findings of Moriondo et al. (2010), who concluded that high yields for southern Europe are expected for spring wheat with an early sowing date, if a long cycle cultivar is used or if supplementary irrigation is applied. In contrast, recovery would be possible with high confidence only for spring wheat under no warming or moderate warming and for slight drying and moderate wetting, or if supplementary irrigation is applied.

In the case of positive adaptation responses, it is notable that EOA values are often higher across a range of climate perturbations for standard maturity cultivars and for spring wheat. Possible explanations of this result include: 1) with standard cycle duration cultivars a shortening cycle, while limiting the grain filling period, also concurrently reduces exposure to water stress, hence minimising overall yield losses, and 2) spring cultivars have no risk of failure due to unfulfilled vernalisation requirements. Other features of EOA outcomes are less straightforward to interpret. For example, low EOA index values were frequently found for simulations in which crops were subjected to stress conditions. It would be necessary to decompose the EOA values for each combination of change producing stress in order to ascertain if they result from a large model spread (perhaps due to differences in the model algorithms representing processes of stress response), or to the nature of the threshold value used to define a positive adaptation response, or to a combination of these.

By assigning an EOA value to every adaptation option, those that are otherwise promising in terms of adaptation response but show low confidence (i.e. low values of EOA) can be discarded. Revisiting the recommendations of Ruiz-Ramos et al. (2018) (who only considered P decreases, see grey-shaded area in Fig. 3) in light of the EOA index generally resulted in narrowing the range for which the adaptation options were effective, more than a dramatic change of the recommended options. As concerning sowing dates, EOA analysis supports the recommendations done in Ruiz-Ramos et al. (2018) for adaptation, while for recovery the main difference was a lower confidence reported by EOA for many cases. For adaptation response, recommendations for standard and longer cultivars were confirmed with very high or maximum confidence, while the confidence level was variable for high perturbations for rainfed spring wheat. For recovery response, results were not modified for winter wheat, but the perturbation range for which spring wheat-based options were effective was smaller than previously estimated. For both adaptation and recovery response, confidence level for shorter cultivars of both winter and spring wheat was lower than for the other cultivars, including some adaptation options previously recommended that now should be excluded (e.g. sw-si-287-cv1 and sw-si-302-cv1, previously recommended for recovery for shallow soil, see Fig. 3 and Fig. S2). As a consequence, the revised recommendations would be to focus on early and standard sowing dates combined with standard and longer cultivars for meeting both adaptation and recovery targets under moderate perturbations with very high confidence, while there would be chances of achieving only adaptation benefit (impact reduction) with these options for severe perturbations. When the aim would be just to adapt, short spring cultivars could also be used with high confidence. The study demonstrates how omitting this analysis would result at least in a number of misleading recommendations under certain perturbations.

An important caveat to attach to all of the above conclusions concerning the potential effectiveness of adaptation measures relates to the use of fixed adjustments (e.g. in sowing dates or in the timing and amount of irrigation) that are applied in conjunction with 30-year means to derive values of EOA. In reality, adjustments in these management practices already take place annually at the present-day, according to seasonal conditions. Applying fixed changes to all years may lead to maladaptation in individual years, unless weather effects in those years are also accounted for (though simulations for the baseline climate are also affected by the same lack of dynamic response to the weather). Whether such maladaptation effects would be accentuated with changes in climate is a matter of conjecture. Simulating such dynamic management responses in conjunction with the fixed adjustments of the type simulated in this study is a modelling requiring the application of weather-based rules that are valid not only in present-day conditions but also across the range of perturbations tested in the IRS and ARS analyses. In the absence of explicit modelling of these effects, the safest way to minimise maladaptation when interpreting our results could be to select adaptation options that have high EOA values for a wide range of perturbations, in an attempt to cover as broad a range of inter-annual variability as possible.

### 4.3 On the use and wider applicability of the EOA index

Finally, it should be noted that the EOA index is intended to evaluate the level of agreement between MME outcomes with respect to a given hypothesis. However, it cannot be used to evaluate differences in the quality of specific ensemble members, nor to detect interdependencies among models. Although in principle a low spread would be expected between models that are related, in our study discrepancies between outcomes from the same models operated by different modelling groups could be greater than those between unrelated models (a finding consistent with Confalonieri et al., 2016). Moreover, the index is also affected by other factors, such as the threshold defined for fulfilling a given hypothesis. Therefore, to ensure the best possible quality of an ensemble, careful pre-selection of models is suggested, based on a diversity of models that reflect the most representative range of model structure and parameterizations possible (Katzav et al., 2012).

Also, EOA index is not intended to be used to rank adaptation options or to identify a “best” option based on numerical EOA values. This is because every adaptation option has been calculated using a different ensemble, and ensemble size and composition affect the results (as we demonstrated in this paper). Besides, EOA values are affected by the choice of the full ensemble. Hence comparisons between their EOA values should be interpreted more carefully. Instead, assessment based on EOA classes could provide this kind of evaluation. Also, EOA calculated for a set of adaptation options provides important information on the relative confidence in their ability to provide adaptation and recovery.

In this study the hypothesis H was defined that the value to be tested should be greater than a given threshold. Minor modifications of the algorithm would be needed to calculate the EOA for responses below a threshold. Another way of assessing ensemble results is to calculate the maximum threshold that obtains a given EOA value. For instance, by setting the EOA index to a high value, it would be possible to identify those positive responses that are estimated with high confidence.

For hypotheses defined using a threshold, another possible modification would be to adopt a double-sided index, providing values in the range [-1, 1], behaving as reported in the examples above for the [0,1] interval (being 0 if the aggregated value matches the threshold), and behaving similarly but with opposite sign if a complementary hypothesis is assumed.

It would also be possible to develop another metric based on the *AF* parameter (see Eq. 2). *AF* is a component of the EOA index, but if it were computed independently for each ensemble size, using the final aggregated value of the full ensemble as a threshold (e.g. median of the adaptation responses), the resulting metric could offer an indicator of spread depicting the internal behaviour of the ensemble for different ensemble sizes and compositions.

Although the EOA index has been developed to work with results from an ensemble of crop simulation models, this approach can be used in other contexts. For instance, it can be used to analyse results of other mechanistic models, not just crop models, or in other situations where observational data are not available with which to compare results (e.g. testing crops not previously grown at a location or ideotypes not yet developed), or to analyse different values of the same magnitude obtained from several measurements or data sources. The approach developed here could be especially useful for data with a wide distribution.

Calculation of the index for a given ensemble is computationally feasible with a reasonable investment of time and resources, at least for the maximum ensemble size considered in this study. For larger ensemble sizes, the index could be calculated using sampling techniques when an exhaustive analysis is not possible (e.g. models with perturbed parameters, see Iizumi et al., 2014; Tao et al., 2017).

## 5 Conclusions

Crop model ensemble size and composition affect the final recommendations concerning adaptation responses to climate change impacts. The ensemble outcome agreement (EOA) index helps to discriminate the recommendations that can be derived from multi-model ensemble outcomes by evaluating their level of confidence. Confidence levels vary depending on the initial set of ensemble members, the adaptation considered, the climate change perturbations assumed, the threshold response fixed for the recommendation and the method of aggregating the results.

Our analysis has demonstrated that effective adaptation of wheat in a Mediterranean environment is feasible with high confidence even for moderate and severe climate perturbations. Spring wheat and supplementary irrigation based options have results with the highest confidence, especially in combination with options that maintain or increase the length of the crop duration. Adaptation enabling maintenance of current yields is also found to be feasible with high confidence under moderate drying and warming for some options involving supplemental irrigation.

The methodology and index defined in this study can be applied effectively to assess confidence levels not only for other multi-model ensembles, but in other contexts too (e.g. climate models, perturbed model parameter experiments, assessing different data sources of the same observations). Providing recommendations to stakeholders that are supported with metrics such as the EOA index should enable them to assess and strengthen their confidence in the potential effectiveness of different adaptation options.
