# Supplementary material for: Implications of crop model ensemble size and composition for estimates of adaptation effects and agreement of recommendations
Source: Agric For Meteorol. 2019 Jan 15;264:351–62. doi: 10.1016/j.agrformet.2018.09.018 (PMC6472678; doi:10.1016/j.agrformet.2018.09.018)
Supplement: Supplementary file 1 [file mmc1.docx]

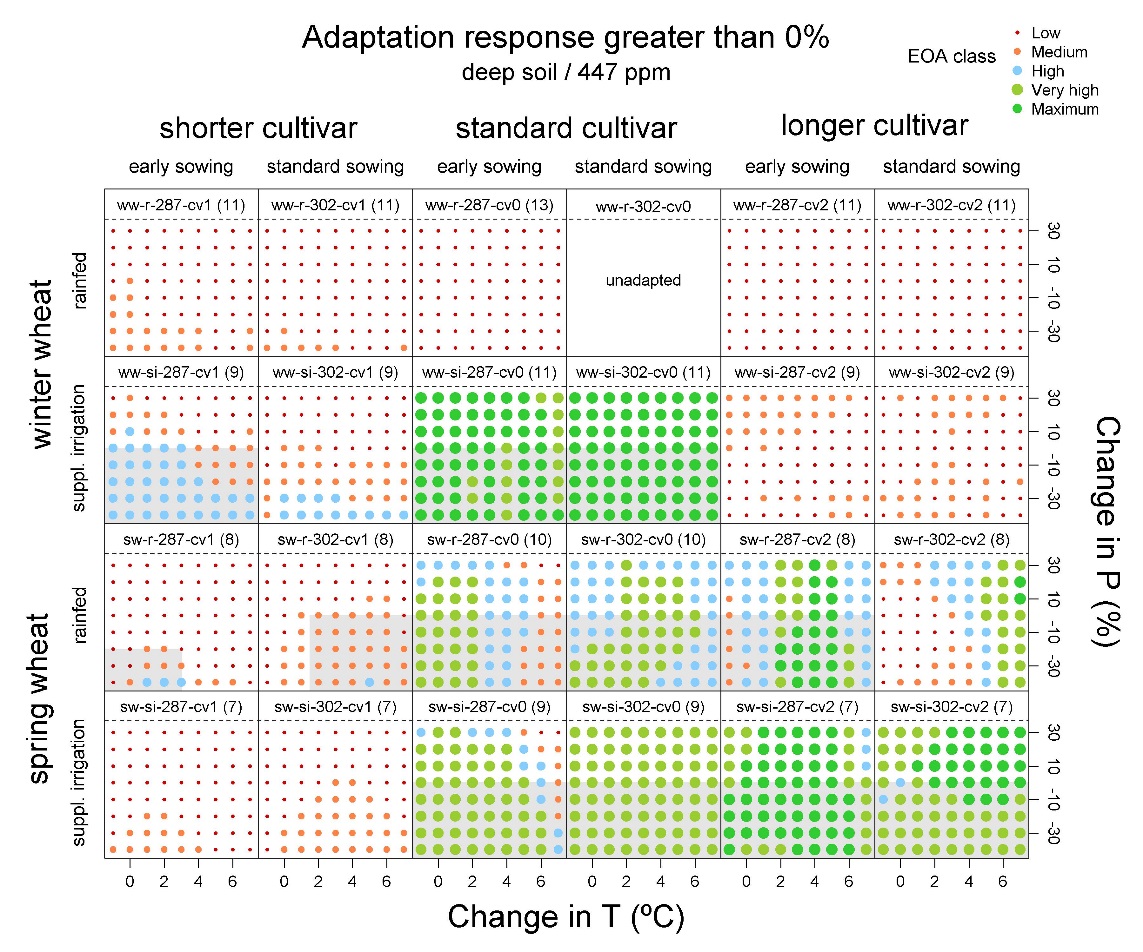


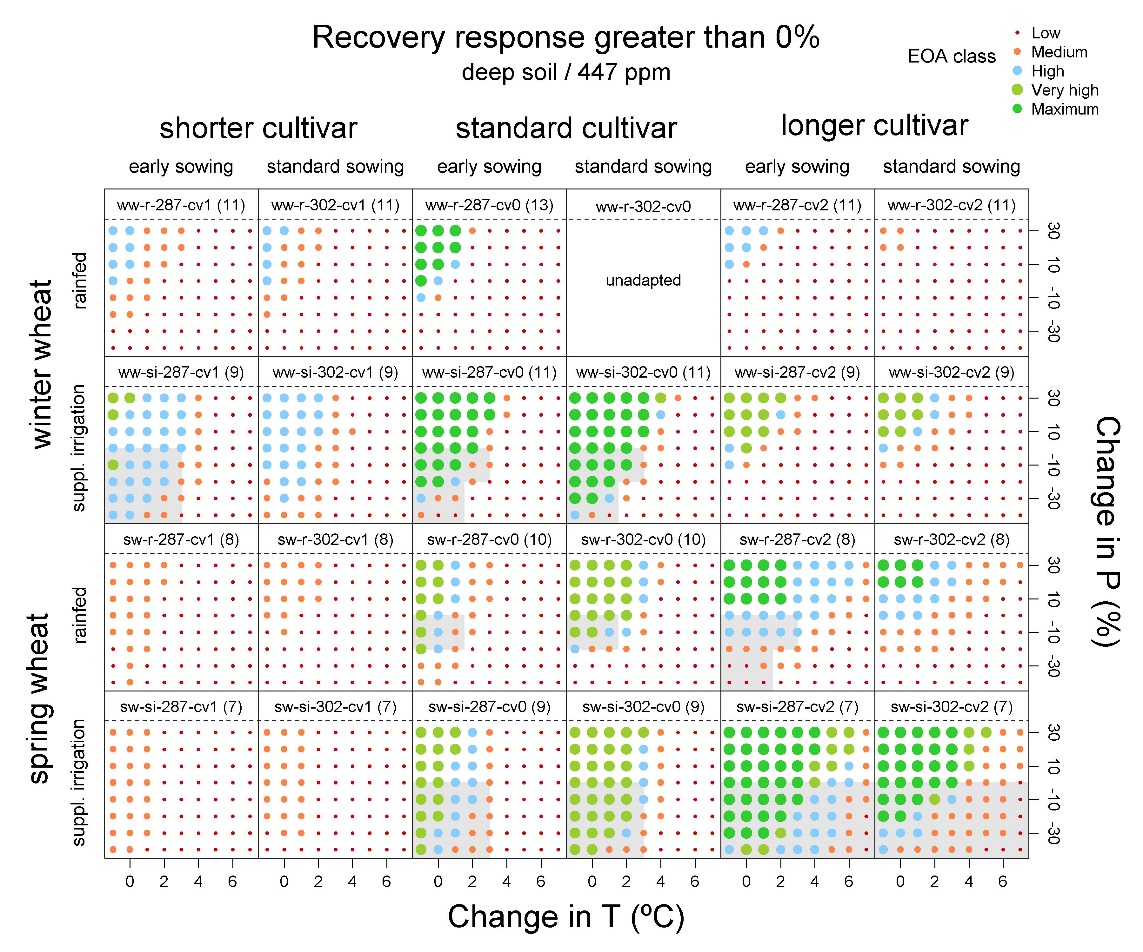


**Fig. S1.** Ensemble outcome agreement (EOA) concerning a positive adaptation response (upper panels) and a positive recovery response (lower panels) for the most promising adaptation options from [Ruiz-Ramos et al. (2018)](#_ENREF_12), assuming deep soil and [CO_2_] of 447 ppm for different temperature (T, ºC) and precipitation (P, %) perturbations. Rows of panels: winter wheat (top two) and spring wheat (bottom two), each for rainfed (upper) and 40 mm of supplementary irrigation applied at anthesis (lower). Columns of panels from left to right are paired by growing duration (10% shorter, standard and 10% longer), each pair alternating between early (DOY, 287) and standard sowing dates (DOY, 302). Grey-shaded areas of each subplot indicate the T and P perturbations for which the adaptation option was recommended in [Ruiz-Ramos et al. (2018)](#_ENREF_12) (P increases not considered). Codes for 23 adaptation options and the unadapted option are described in Table S2 and shown in panel headers with the number of ensemble members used in parentheses. EOA classes are described in Table 1.


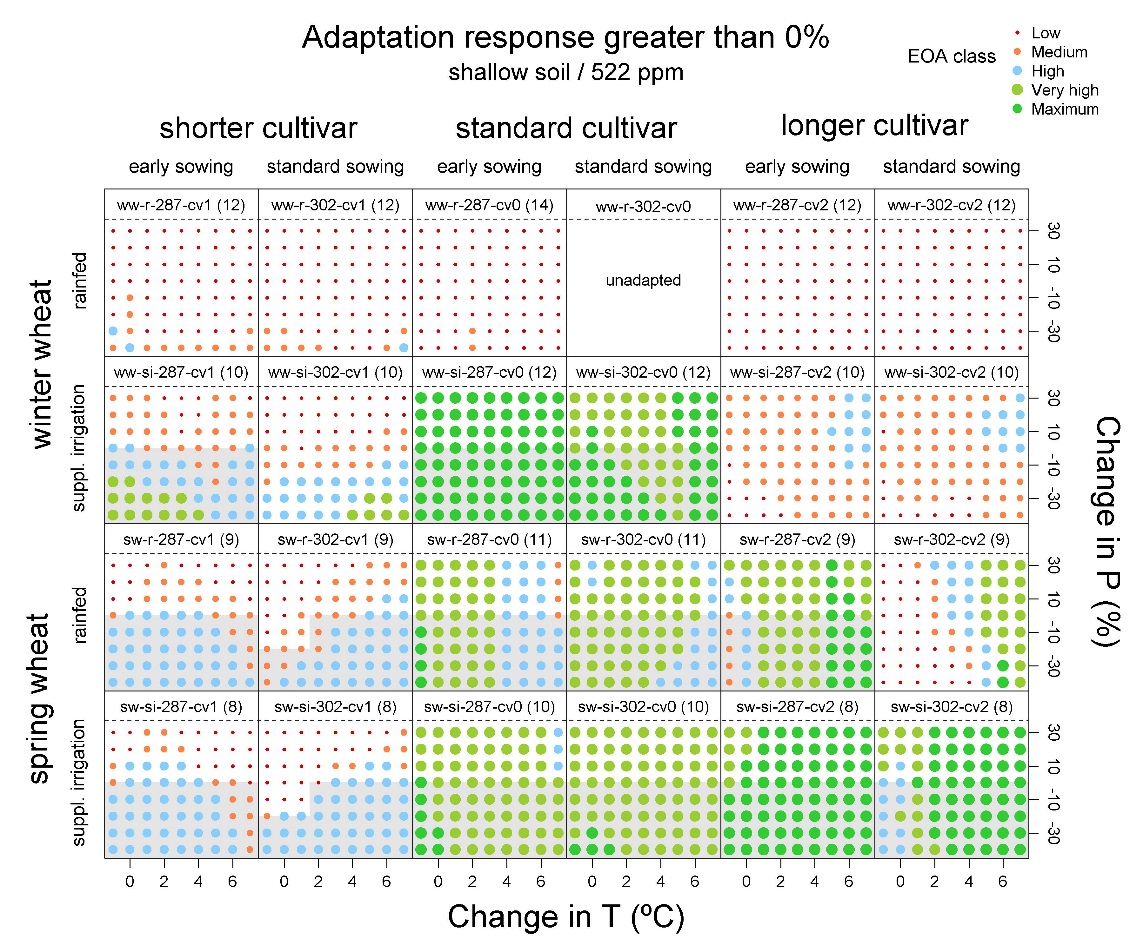


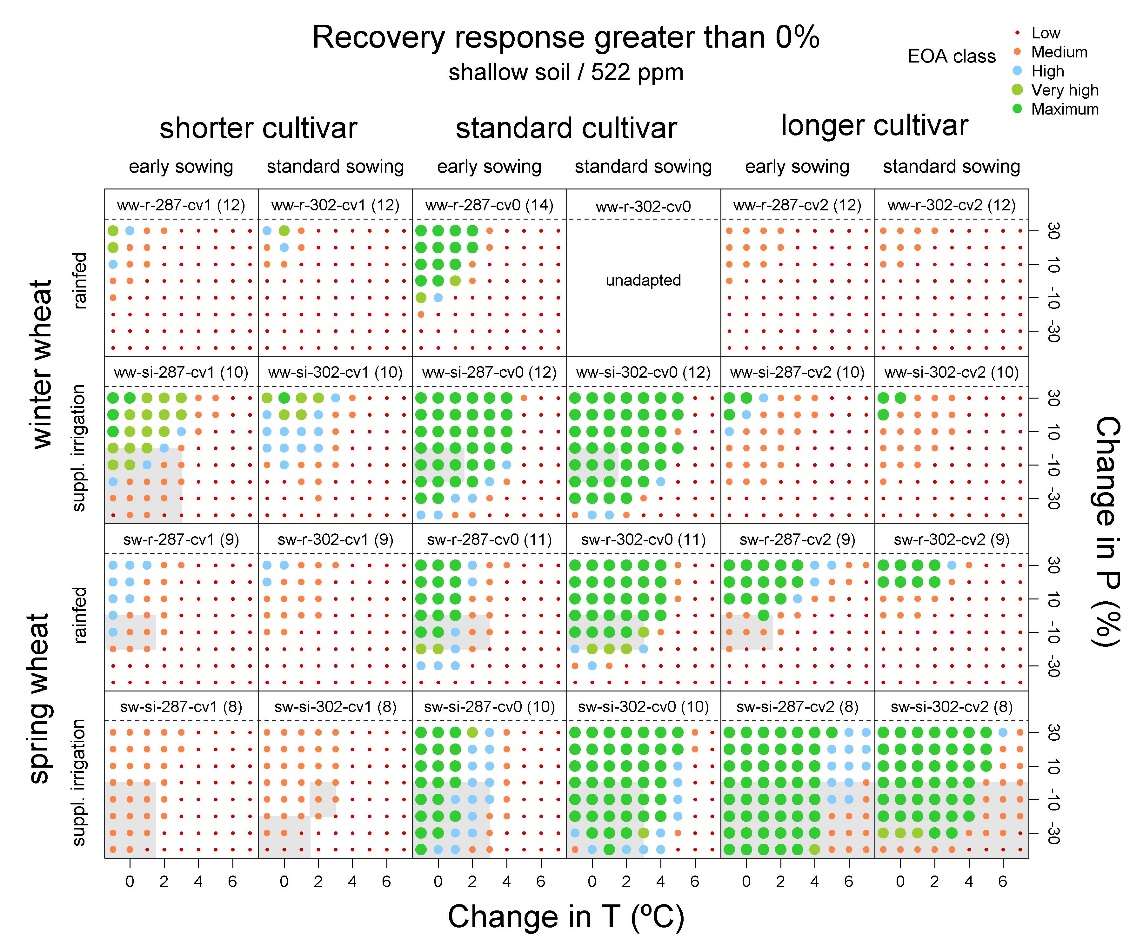


**Fig. S2.** As Fig. S1 but for shallow soil and 522 ppm of [CO_2_]


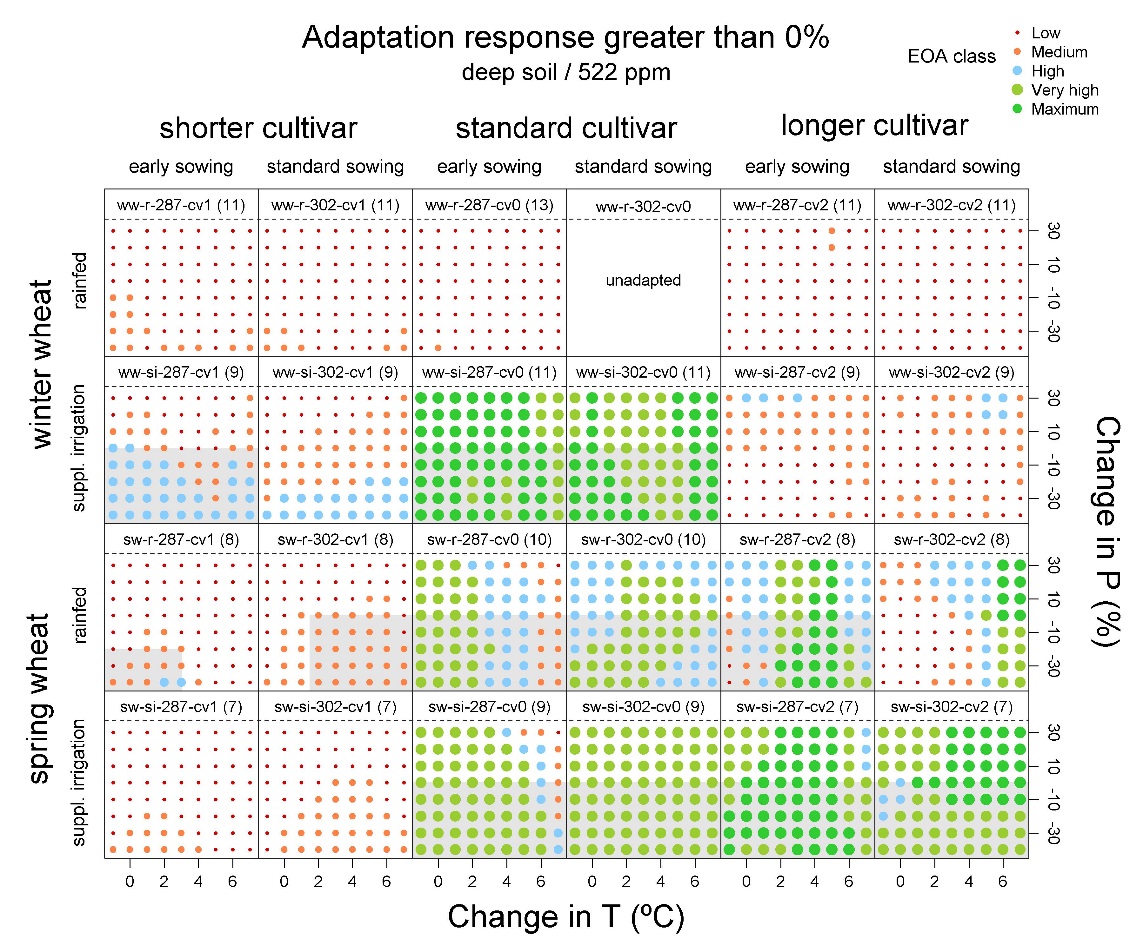


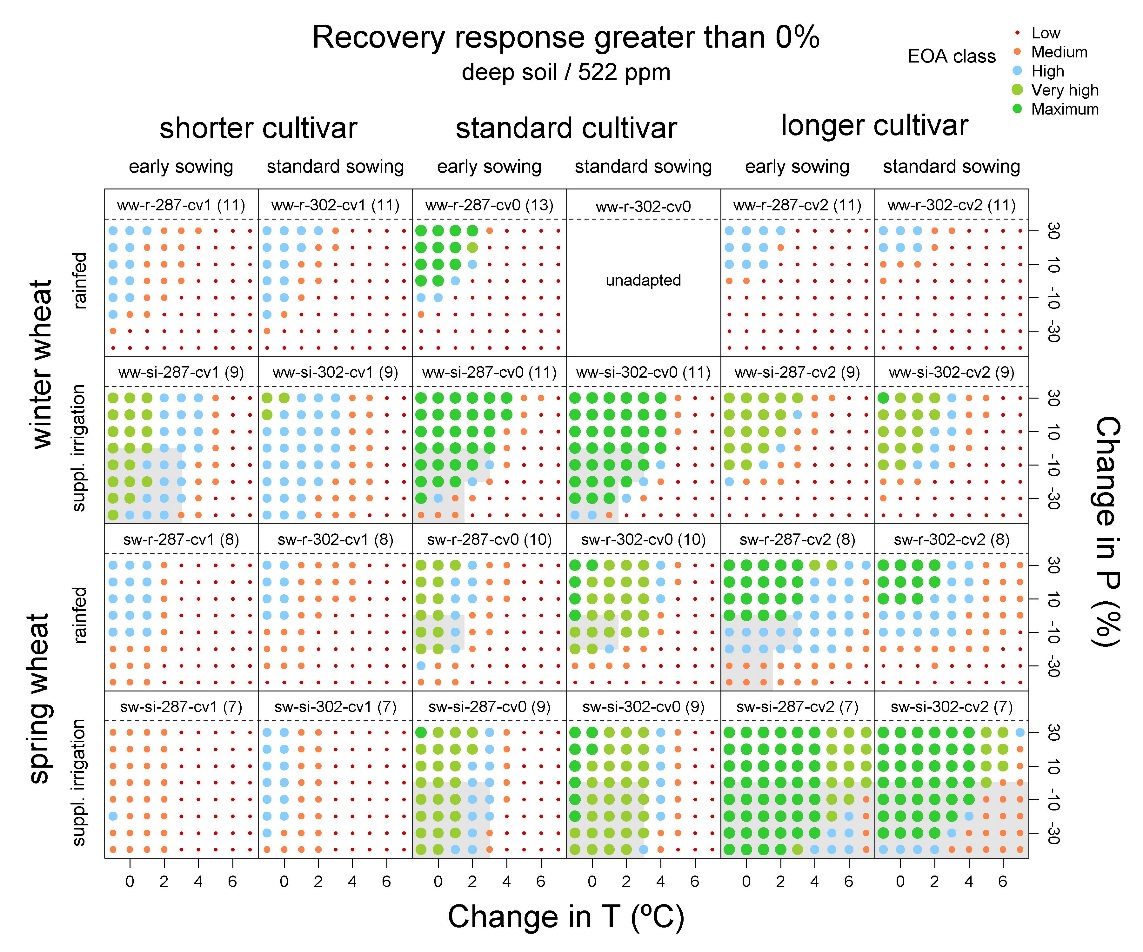


**Fig. S3.** As Fig. S2 but for deep soil.


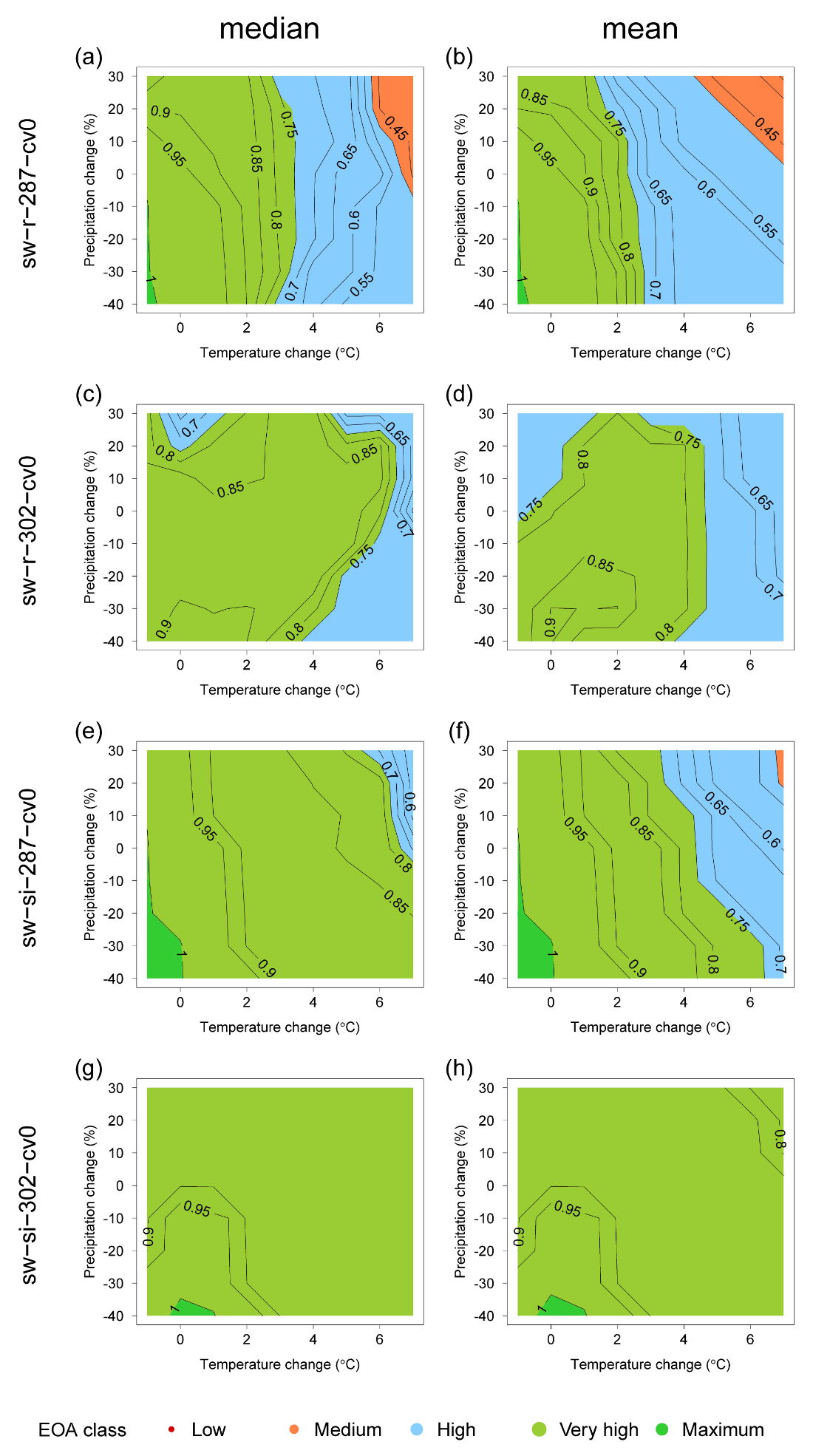


**Fig. S4.** Comparison of EOA response surfaces computed for median (left column) and mean (right column) multi-model ensemble adaptation responses assuming three promising adaptation options: rainfed spring wheat for early sowing date and standard duration cultivar (sw-r-287-cv0, top row); rainfed spring wheat for standard sowing date and standard duration cultivar (sw-r-302-cv0, second row); irrigated spring wheat for early sowing date and standard duration cultivar (sw-si-287-cv0, third row); irrigated spring wheat for standard sowing date and standard duration cultivar (sw-si-302-cv0, bottom row). See Table 1 for interpreting EOA classes. Codes for adaptation options are described in Table S2.

**Table S1**

List of wheat models applied in the study. Each model was used by one modellers group excepting those marked with a footnote indicating the number of groups that provided model results for the model, each one from a different calibration (see [Ruiz-Ramos et al., 2018](#_ENREF_12)).

| **Model** | **Reference** | **Web documentation** |
| --- | --- | --- |
| AFRCWHEAT2 | [Porter (1984)](#_ENREF_11) | - |
| CARAIB | [Dury et al. (2011)](#_ENREF_4) | [www.umccb.ulg.ac.be/Sci/m_car_e.html](http://www.umccb.ulg.ac.be/Sci/m_car_e.html) |
| CERES-wheat DSSAT v.4.5^a^ | [Jones et al. (2003)](#_ENREF_6) | http://dssat.net |
| DNDC v.9.5 | [Deng et al. (2011)](#_ENREF_3) | [www.dndc.sr.unh.edu](http://www.dndc.sr.unh.edu/) |
| HERMES v.4.26 | [Kersebaum and Nendel (2014)](#_ENREF_7) | www.zalf.de/en/forschung_lehre/software_downloads/ |
| Lintul4 | [Shibu et al. (2010)](#_ENREF_14) | http://models.pps.wur.nl/node/950 |
| LPJ-GUESS | [Lindeskog et al. (2013)](#_ENREF_8) | <http://iis4.nateko.lu.se/lpj-guess> |
| MCWLA-Wheat model | [Tao and Zhang (2013)](#_ENREF_15) | - |
| MONICA | [Nendel et al. (2011)](#_ENREF_10) | http://monica.agrosystem-models.com/en |
| SIMPLACE<Lintul2,Slim> | [Gaiser et al. (2013)](#_ENREF_5) | [www.simplace.net](http://www.simplace.net/) |
| Sirius 14 | [Semenov et al. (2014)](#_ENREF_13) | http://resources.rothamsted.ac.uk/mas-models/sirius |
| SiriusQuality2 | [Martre et al. (2006)](#_ENREF_9) | www1.clermont.inra.fr/siriusquality |
| STICS v.8.4.1 | [Brisson et al. (2003)](#_ENREF_2) | www6.paca.inra.fr/stics |
| WOFOST v.7.1^b^ | [Boogaard et al. (2013)](#_ENREF_1) | www.wofost.wur.nl |

^a^ Used by two modeller groups

^b^ Used by three modeller groups

**Table S2**

List of codes for adaptation options and descriptions. Every code has four components: cultivar type, water management, sowing dates and cycle duration. When one (or more) component is not specified, standard option is assumed (some examples of abbreviated codes are provided in the central column; e.g. sw-si is equivalent to sw-si-302-cv0).

| **Full code** | **Examples of abbreviated codes** | **Description** |
| --- | --- | --- |
| ww-r-302-cv0^a^ | ww, r, 302, cv0, ww-cv0, ww-302 | Winter wheat, rainfed, standard sowing date (302, DOY), standard cultivar duration |
| ww-r-302-cv1 | cv1, ww-cv1 | Winter wheat, rainfed, standard sowing date (302, DOY), 10% shorter cultivar duration |
| ww-r-302-cv2 | cv2, ww-cv2 | Winter wheat, rainfed, standard sowing date (302, DOY), 10% longer cultivar duration |
| ww-r-287-cv0 | 287, ww-287 | Winter wheat, rainfed, early sowing date (287, DOY), standard cultivar duration |
| ww-r-287-cv1 | 287-cv1, ww-287-cv1 | Winter wheat, rainfed, early sowing date (287, DOY), 10% shorter cultivar duration |
| ww-r-287-cv2 | 287-cv2, ww-287-cv2 | Winter wheat, rainfed, early sowing date (287, DOY), 10% longer cultivar duration |
| ww-si-302-cv0 | si, ww-si | Winter wheat, application of supplementary irrigation (40 mm) at flowering, standard sowing date (302, DOY), standard cultivar duration |
| ww-si-302-cv1 | si-cv1, ww-si-cv1 | Winter wheat, application of supplementary irrigation (40 mm) at flowering, standard sowing date (302, DOY), 10% shorter cultivar duration |
| ww-si-302-cv2 | si-cv2, ww-si-cv2 | Winter wheat, application of supplementary irrigation (40 mm) at flowering, standard sowing date (302, DOY), 10% longer cultivar duration |
| ww-si-287-cv0 | si-287, ww-si-287 | Winter wheat, application of supplementary irrigation (40 mm) at flowering, early sowing date (287, DOY), standard cultivar duration |
| ww-si-287-cv1 | si-287-cv1 | Winter wheat, application of supplementary irrigation (40 mm) at flowering, early sowing date (287, DOY), 10% shorter cultivar duration |
| ww-si-287-cv2 | si-287-cv2 | Winter wheat, application of supplementary irrigation (40 mm) at flowering, early sowing date (287, DOY), 10% longer cultivar duration |
| sw-r-302-cv0 | sw, sw-302 | Spring wheat, rainfed, standard sowing date (302, DOY), standard cultivar duration |
| sw-r-302-cv1 | sw-cv1, sw-302-cv1 | Spring wheat, rainfed, standard sowing date (302, DOY), 10% shorter cultivar duration |
| sw-r-302-cv2 | sw-cv2, sw-302-cv2 | Spring wheat, rainfed, standard sowing date (302, DOY), 10% longer cultivar duration |
| sw-r-287-cv0 | sw-287, sw-287-cv0 | Spring wheat, rainfed, early sowing date (287, DOY), standard cultivar duration |
| sw-r-287-cv1 | sw-287-cv1 | Spring wheat, rainfed, early sowing date (287, DOY), 10% shorter cultivar duration |
| sw-r-287-cv2 | sw-287-cv2 | Spring wheat, rainfed, early sowing date (287, DOY), 10% longer cultivar duration |
| sw-si-302-cv0 | sw-si, sw-si-302 | Spring wheat, application of supplementary irrigation (40 mm) at flowering, standard sowing date (302, DOY), standard cultivar duration |
| sw-si-302-cv1 | sw-si-cv1 | Spring wheat, application of supplementary irrigation (40 mm) at flowering, standard sowing date (302, DOY), 10% shorter cultivar duration |
| sw-si-302-cv2 | sw-si-cv2 | Spring wheat, application of supplementary irrigation (40 mm) at flowering, standard sowing date (302, DOY), 10% longer cultivar duration |
| sw-si-287-cv0 | sw-si-287 | Spring wheat, application of supplementary irrigation (40 mm) at flowering, early sowing date (287, DOY), standard cultivar duration |
| sw-si-287-cv1 | - | Spring wheat, application of supplementary irrigation (40 mm) at flowering, early sowing date (287, DOY), 10% shorter cultivar duration |
| sw-si-287-cv2 | - | Spring wheat, application of supplementary irrigation (40 mm) at flowering, early sowing date (287, DOY), 10% longer cultivar duration |

DOY: day of the year.

^a^ Standard option.

**References**

Boogaard, H., Wolf, J., Supit, I., Niemeyer, S. and van Ittersum, M., 2013. A regional implementation of WOFOST for calculating yield gaps of autumn-sown wheat across the European Union. Field Crop. Res., 143: 130-142.

Brisson, N. et al., 2003. An overview of the crop model STICS. Eur. J. Agron., 18(3-4): 309-332.

Deng, J. et al., 2011. Modeling nitrogen loading in a small watershed in southwest China using a DNDC model with hydrological enhancements. Biogeosciences, 8(10): 2999-3009.

Dury, M. et al., 2011. Responses of European forest ecosystems to 21st century climate: Assessing changes in interannual variability and fire intensity. IForest, 4: 82-99.

Gaiser, T. et al., 2013. Modeling biopore effects on root growth and biomass production on soils with pronounced sub-soil clay accumulation. Ecol. Model., 256: 6-15.

Jones, J.W. et al., 2003. The DSSAT cropping system model. Eur. J. Agron., 18(3–4): 235-265.

Kersebaum, K.C. and Nendel, C., 2014. Site-specific impacts of climate change on wheat production across regions of Germany using different CO2 response functions. Eur. J. Agron., 52: 22-32.

Lindeskog, M. et al., 2013. Implications of accounting for land use in simulations of ecosystem carbon cycling in Africa. Earth Syst. Dynam., 4(2): 385-407.

Martre, P. et al., 2006. Modelling protein content and composition in relation to crop nitrogen dynamics for wheat. Eur. J. Agron., 25(2): 138-154.

Nendel, C. et al., 2011. The MONICA model: Testing predictability for crop growth, soil moisture and nitrogen dynamics. Ecol. Model., 222(9): 1614-1625.

Porter, J.R., 1984. A model of canopy development in winter wheat. J. Agric. Sci., 102(2): 383-392.

Ruiz-Ramos, M. et al., 2018. Adaptation response surfaces for managing wheat under perturbed climate and CO2 in a Mediterranean environment. Agric. Syst., 159: 260-274.

Semenov, M.A., Stratonovitch, P., Alghabari, F. and Gooding, M.J., 2014. Adapting wheat in Europe for climate change. J. Cereal Sci., 59(3): 245-256.

Shibu, M.E., Leffelaar, P.A., van Keulen, H. and Aggarwal, P.K., 2010. LINTUL3, a simulation model for nitrogen-limited situations: Application to rice. Eur. J. Agron., 32(4): 255-271.

Tao, F. and Zhang, Z., 2013. Climate change, wheat productivity and water use in the North China Plain: A new super-ensemble-based probabilistic projection. Agric. For. Meteorol., 170: 146-165.
